# High Serum AST/ALT Ratio and Low Serum INS*PA Product Are Risk Factors and Can Diagnose Sarcopenia in Middle-Aged and Older Adults

**DOI:** 10.3389/fendo.2022.843610

**Published:** 2022-03-18

**Authors:** Yong He, Fing Ding, Mengting Yin, He Zhang, Lisha Hou, Tao Cui, Jinfeng Xu, Jirong Yue, Qin Zheng

**Affiliations:** ^1^Department of Laboratory Medicine, West China Hospital, Sichuan University, Chengdu, China; ^2^Department of Geriatrics and National Clinical Research Center for Geriatrics, West China Hospital, Sichuan University, Chengdu, China; ^3^Department of Gynecology and Obstetrics, West China Second University Hospital, Sichuan University, Chengdu, China; ^4^Key Laboratory of Birth Defects and Related Diseases of Women and Children (Sichuan University), Ministry of Education, Chengdu, China

**Keywords:** sarcopenia, middle-aged and older, alanine aminotransferase, insulin, prealbumin

## Abstract

**Background:**

Sarcopenia is an age-related clinical condition and associated with an increased risk of adverse outcomes. However, to date, there is no global standard for the diagnosis of sarcopenia, and fewer serum biomarkers have been suggested for the diagnosis of sarcopenia. It is, thus, important that sarcopenia-related serological diagnostic markers be explored. The present study was based on the Asian Working Group on Sarcopenia 2019 (AWGS 2019) criteria to assess whether aspartate aminotransferase/alanine aminotransferase (AST/ALT) ratio and fasting insulin*prealbumin (INS*PA) product are diagnostic markers associated with sarcopenia in various ethnic groups in western China.

**Methods:**

This cross-sectional study included 4,099 adults (1,471 men and 2,628 women) from the West China Health and Aging Trend (WCHAT) study. The value of serum biomarkers was based on laboratory data. The accompanying metabolic disorders and the associated parameters were evaluated. Logistic regression analysis was used to explore the association between markers and sarcopenia. Receiver operating characteristic curve (ROC) analysis was used to evaluate the diagnostic efficacy of the test in differentiating sarcopenia.

**Results:**

Binary regression analysis showed that high serum AST/ALT (OR = 2.247) and adrenal cortisol (PTC, OR = 1.511), low serum INS*PA (OR = 2.970), free triiodothyronine (FT3, OR = 1.313), 25-OH-VitD (VitD, in male participants, OR = 1.817), and diastolic blood pressure (DBP, in female subjects, OR = 1.250) were independent risk factors for sarcopenia (*P* < 0.05). AST/ALT and INS*PA were not affected by metabolic factors and had better diagnostic efficacy for sarcopenia. The AUC of the INS*PA was the highest (0.705, 0.706, and 0.701, respectively, *P* < 0.05), followed by that of the AST/ALT (0.680, 0.675, and 0.695, respectively, *P* < 0.05). The AUC of the AST/ALT/(INS*PA)*10,000 used to diagnose sarcopenia was 0.727.

**Conclusion:**

Among middle-aged and older adults of multiple ethnicities in western China, we found that higher AST/ALT and lower INS*PA levels are associated with an increased prevalence of sarcopenia. Since these serum biomarkers are inexpensive and can be obtained easily from biochemical routine, regular follow-up of AST/ALT and INS*PA may be an effective strategy in sarcopenia screening and management.

## Introduction

Sarcopenia is an age-related clinical condition characterized by progressive loss of skeletal muscle mass with reduced muscle strength and physical performance (1). The problems of the elderly are attracting greater attention largely because the global population is steadily growing older as the proportion of its aged members increases. In 2019, the Chinese population constituted 18% of the world population, with 164.5 million Chinese citizens aged 65 and above (65+) (2). A study, according to the Asian Working Group on Sarcopenia (AWGS), reported that the prevalence rate of sarcopenia in the Chinese population over 60 years old was 10.5% (3). Age, physical inactivity, insulin resistance, endocrine factors, and low-grade chronic inflammation are the main mechanisms underlying sarcopenia (4).

Sarcopenia is associated with an increased risk of adverse outcomes and is reported to have approximately two‐ to four‐fold higher risk of mortality and functional disability irrespective of differences in the measurement and criteria of sarcopenia (5, 6). The prevalence of sarcopenia increases with age; however, it can be treated and even cured (7). Early diagnosis and intervention are extremely important to arrest the disability cascade that accompanies a clinical condition with this potential prevalence and well-known negative outcomes (8).

Several studies have reported laboratory biomarkers for the diagnosis of sarcopenia. One study reported that low alanine aminotransferase (ALT) levels predict low muscle strength in older patients with diabetes (9). ALT is a transaminase enzyme found in the liver and muscle tissue (10), while aspartate aminotransferase (AST) is expressed in the liver, heart, skeletal muscle, etc. Generally, high ALT or AST values (>40 IU/L) are considered pathological and reflect liver damage, caused by hepatitis, etc. (11). Another study also reported that low ALT activity in the peripheral blood is a surrogate marker for low general body muscle mass and sarcopenia (12). The aspartate aminotransferase/alanine aminotransferase (AST/ALT) ratio has been reported to be associated with mortality and disability in chronic liver disease, which is an age-related disease characterized by chronic low-grade inflammation (13). So, its role as a possible diagnostic biomarker for sarcopenia with the same pathogenesis of chronic low-grade inflammation was evaluated.

Prealbumin (PA), another biomarker, has been reported that its levels were inverse with sarcopenia incidence in hospitalized patients over 60 years old (14) or in older men with type 2 diabetes mellitus (15). PA is an indicator of protein metabolic balance in a non-acutely infected individual, which has a short half-life and can reflect drastic changes in nutritional status (16). Meanwhile, insulin plays a major role in protein metabolism by stimulating and enhancing protein synthesis and inhibiting proteolysis (17). A number of studies have reported that hyperinsulinemia can increase muscle protein synthesis (18). We used fasting insulin*prealbumin (INS*PA) to amplify the difference between sarcopenia and non-sarcopenia groups and to evaluate its potential as a diagnostic biomarker for sarcopenia. To our knowledge, this is the first study to report the effectiveness of the INS*PA product in the diagnosis of sarcopenia.

The serological method is cheap and simple for the diagnosis of sarcopenia, and there is no universal diagnostic standard for sarcopenia. Through this study, we hope to find the calculated value of some combination of biomarkers to amplify the difference between the sarcopenia group and the non-sarcopenia group, so as to better assist in the prediction of sarcopenia. The aim of this study was to evaluate the relationship between AST/ALT, INS*PA, and sarcopenia among elderly Chinese participants from the West China Health and Aging Trend (WCHAT) study under the background of China’s aging population. Moreover, we evaluate whether it is confounded by other metabolic factors in older participants. In addition, we calculate the usefulness of AST/ALT and INS*PA in predicting sarcopenia among all participants, male participants, and female participants, respectively.

## Methods

### Data Collection

The current research was a cross-sectional analysis of baseline data from the WCHAT study (19), which was initiated in 2018 and included 7,536 people aged 50 or older in west China. Our sampling method was multistage cluster sampling with a response rate of 50.2% in baseline data collection. The exclusion criteria were as follows: participants who were not assessed for sarcopenia or lacked basic information or data on the laboratory biomarkers included in our study and participants diagnosed with mental illness, tumors, or chronic kidney or liver disease. Finally, a total of 4,099 subjects aged 50 or older from various communities in southwest China’s Sichuan province were enrolled in the study, of which 780 patients were diagnosed with sarcopenia. This study was approved by the Ethics Committee of the West China Hospital of Sichuan University (reference: 2017-445) and was carried out in accordance with the approved guidelines.

### Assessment of Sarcopenia

Appendix skeletal muscle mass (ASM) was measured using the InBody 770 instrument (BioSpace, Seoul, South Korea) by bioelectrical impedance analysis (BIA). We calculated the ASM index (ASMI, ASM/height^2^). Handgrip strength was determined twice by a myometer (EH101; Camry, Zhongshan, China) and we recorded the maximum. Physical performance was evaluated by the 6-m habitual walking speed test, in which participants were asked to walk at their usual speed. We used the AWGS 2019 as the diagnostic criteria, considering both a low muscle mass (ASMI, men: <7.0 kg/m^2^ and women: <5.7 kg/m^2^) and a low muscle strength (handgrip strength, men: <28 kg, women: <18 kg) or a low level of physical performance (6 m walking test < 1 m/s).

### Data Collection and Laboratory Analysis

Interviewers were students from the West China Clinical Medical College of Sichuan University, who collected questionnaire data through face-to-face, one-on-one personal interviews. Trained technicians performed the anthropometric and bioimpedance measurements. Demographic factors related to individual study subjects were recorded, including age, sex, smoking history, alcohol drinking history, number of chronic diseases, activity of daily living (ADL), height and weight, body mass index (BMI, weight/height^2^), and blood pressure (BP, tested twice).

We collected specimens from the antecubital vein after an overnight fast. Biomarkers including thyroid stimulating hormone (TSH), FT3, free tetraiodothyronine (FT4), fasting insulin (INS), fasting blood glucose (GLU), total protein (TP), PA, albumin (ALB), ALT, AST, creatinine (CREA), urea nitrogen (UREA), uric acid (UA), triglycerides (TG), total cholesterol (CHOL), high-density lipoprotein (HDL), low-density lipoprotein (LDL), very LDL (VLDL), VitD, white blood cell (WBC), neutrophil ratio (NPR), lymphocyte ratio (LPR), platelet (PLT), and PTC were measured. INS*PA is the product of INS and PA, AST/ALT is the ratio of AST and ALT, and globulin (GLO) is the difference of TP minus ALB.

NPR, LPR, and PLT were used with whole blood (Medonic CA620, Sweden), except WBC, and the remaining blood was centrifuged at 3,000 rpm for 10 min to obtain serum. TSH, FT3, FT4, and INS were measured using the Roche Cobas e411 analyzer (Germany); GLU, TP, PA, ALB, ALT, AST, CREA, UREA, UA, TG, CHOL, HDL, LDL, and VLDL were measured using the Olympus AU400 analyzer (Japan); VitD was measured using the Abbott i2000SR analyzer (USA); and PTC was measured using the Siemens Centaur XP analyzer (Germany).

Hypertension was defined as systolic BP (SBP) ≥140 mmHg or DBP ≥90 mmHg or taking a hypertensive drug. Obesity was defined as BMI ≥25 kg/m^2^. Hypertriglyceridemia was defined as TG levels ≥2.26 mmol/L. Diabetes mellitus was defined as GLU levels ≥7.0 mmol/L or being previously diagnosed as having diabetes by a physician.

### Statistical Analyses

The boxplot was graphed using GraphPad Prism 8. The bar chart and trend line were generated using Excel 2016. The receiver operating characteristic (ROC) curve analysis and the ROC comparison analysis of MedCalc v19.0.7 were used to evaluate the diagnostic efficacy of the test in differentiating sarcopenia. The Kolmogorov–Smirnov test was used to estimate data distribution. Non-normally distributed continuous variables were presented as medians and values of 25 to 75 percentiles, and categorical variables were presented as frequencies with percentages. The Mann–Whitney *U* test was used to compare biomarker levels between sarcopenia and non-sarcopenia, and the chi-square test was used for the analysis of categorical variables. Binary regression analysis was used to estimate risk factors for sarcopenia. Variables with statistical differences between groups were selected for the binary regression model. When collinear features existed between two variables, either of the two variables was removed from the multiple regression model. The criterion for binary regression diagnosis was the median of all participants. All other statistical analyses were done using SPSS 19.0. P *<*0.05 was considered statistically significant.

## Results

### Baseline Characteristics of the Study Cohorts Stratified by Sarcopenia

Among the 4,099 elderly participants, 780 had sarcopenia (325 men, 455 women), whereas 3,319 did not have sarcopenia (1,146 men, 2,173 women). **Table 1** shows the baseline characteristics of the elderly participants stratified by sarcopenia. Compared with the non-sarcopenia group, the sarcopenia group was significantly older, had more male participants, and a higher proportion of smoking history, number of chronic diseases, and ADL disability. The sarcopenia group had a lower level of BMI, SBP, and DBP than the non-sarcopenia group. The proportion of alcohol drinking history was not different between the two groups.

**Table 1:** Baseline characteristics of the elderly participants stratified by sarcopenia.

	No sarcopenia (*n* = 3319)	Sarcopenia (*n* = 780)	*P-*value
Age (years)	61 (54, 66)	68 (61, 74)	<0.001
Sex (*n*, %)			<0.001
Male	1,146 (34.5)	325 (41.7)	
Female	2,173 (65.5)	455 (58.3)	
Smoking history (*n*, %)			<0.001
No	2,771 (83.5)	599 (76.8)	
Yes	548 (16.5)	181 (23.2)	
Alcohol drinking history (*n*, %)			0.115
No	2,456 (74.0)	594 (76.2)	
Yes	863 (26.0)	186 (23.8)	
Number of chronic diseases (*n*, %)			<0.001
0	2,174 (65.5)	513 (65.7)	
1	830 (25.5)	179 (22.9)	
≥2	315 (9.0)	88 (11.4)	
ADL (*n*, %)			<0.001
Normal	2,954 (89.0)	655 (84.0)	
ADL disability	365 (11.0)	125 (16.0)	
BMI (kg/m^2^)	25.81 (23.58, 28.19)	21.91 (20.15, 23.71)	<0.001
Blood pressure (mmHg)			
SBP	128 (115, 142)	126 (113, 140)	0.014
DBP	81 (74, 89)	79 (71, 86)	<0.001

ADL, activity of daily living; SBP, systolic blood pressure; DBP, diastolic blood pressure.

### Metabolic Characteristics of the Study Cohorts Stratified by Sarcopenia

**Table 2** shows the metabolic characteristics of elderly participants stratified by the presence or absence of sarcopenia. Compared with non-sarcopenia patients, those with sarcopenia had a significantly lower FT3, INS, GLU, TP, PA, ALB, ALT, CREA, UA, TG, VLDL, VitD concentration, INS*PA, and LPR (*P* = 0.034 for CREA; *P* = 0.001 for VitD; *P* < 0.001 for the other biomarkers). Compared with patients without sarcopenia, those with sarcopenia had a significantly higher AST/ALT, serum FT4, HDL, PTC concentration, and NPR (*P* < 0.001). There were no differences between the groups with and without sarcopenia regarding TSH, GLO, AST, UREA, CHOL, LDL, WBC, and PLT concentration.

**Table 2:** The metabolic characteristics of elderly participants stratified by sarcopenia.

Metabolic characteristics	No sarcopenia (*n* = 3,319)	Sarcopenia (*n* = 780)	*P-*value
TSH (mIU/L)	2.82 (1.84, 4.26)	2.67 (1.72, 4.19)	0.124
FT3 (pmol/L)	4.51 (4.11, 4.95)	4.34 (3.96, 4.82)	<0.001
FT4 (pmol/L)	17.80 (16.10, 19.54)	18.27 (16.39, 20.17)	<0.001
INS (μU/ml)	7.30 (4.99, 10.64)	4.73 (3.20, 7.15)	<0.001
GLU (mmol/L)	5.18 (4.82, 5.70)	5.01 (4.65, 5.49)	<0.001
TP (g/L)	71.50 (68.50, 74.80)	70.70 (67.40, 74.08)	<0.001
PA (g/L)	272.00 (241.00, 305.00)	253.00 (221.00, 287.75)	<0.001
ALB (g/L)	44.40 (42.50, 46.20)	43.20 (41.20, 45.40)	<0.001
GLO (g/L)	27.30 (24.70, 30.10)	27.60 (25.10, 30.00)	0.077
INS*PA (μU/ml*g/L)	1,991.76 (1,293.96, 3,057.62)	1,191.54 (776.71, 1,867.10)	<0.001
ALT (U/L)	24 (18, 33)	19 (15, 27)	<0.001
AST (U/L)	26 (22, 32)	27 (22, 33)	0.446
AST/ALT	1.10 (0.88, 1.35)	1.38 (1.09, 1.70)	<0.001
CREA (μmol/L)	78.30 (71.00, 87.80)	77.20 (69.40, 86.70)	0.034
UREA (mmol/L)	5.16 (4.29, 6.24)	5.29 (4.28, 6.37)	0.171
UA (μmol/L)	320.30 (274.00, 382.20)	308.95 (259.23, 366.33)	<0.001
TG (mmol/L)	1.46 (1.00, 2.18)	1.27 (0.90, 1.78)	<0.001
CHOL (mmol/L)	4.73 (4.19, 5.32)	4.68 (4.13, 5.29)	0.18
HDL (mmol/L)	1.23 (1.04, 1.42)	1.32 (1.11, 1.54)	<0.001
LDL (mmol/L)	2.69 (2.20, 3.21)	2.66 (2.16, 3.16)	0.319
VLDL (mmol/L)	0.66 (0.45, 0.99)	0.58 (0.41, 0.81)	<0.001
VitD (ng/ml)	18.60 (14.80, 23.10)	17.80 (14.10, 22.20)	0.001
WBC (10^9^/L)	5.60 (4.80, 6.60)	5.70 (4.80, 6.70)	0.28
NPR (%)	61.10 (55.60, 66.40)	62.20 (56.20, 68.60)	<0.001
LPR (%)	31.40 (26.50, 36.70)	30.30 (24.50, 36.10)	<0.001
PLT (10^9^/L)	164 (129, 203)	167 (127, 205)	0.833
PTC (nmol/L)	328.90 (242.90, 429.60)	367.45 (265.85, 465.33)	<0.001

TSH, thyroid-stimulating hormone; FT3, free triiodothyronine; FT4, free tetraiodothyronine; INS, fasting insulin; GLU, fasting blood glucose; TP, total protein; PA, prealbumin; ALB, albumin; GLO, globulin; INS*PA, product of INS and PA; ALT, alanine aminotransferase; AST, aspartate aminotransferase; AST/ALT, AST to ALP ratio; CREA, creatinine; UREA, urea nitrogen; UA, uric acid; TG, triglycerides; CHOL, total cholesterol; HDL, high-density lipoprotein; LDL, low-density lipoprotein; VLDL, very LDL; VitD, 25-OH-VitD; WBC, white blood cell; NPR, neutrophil ratio; LPR, lymphocyte ratio; PLT, platelet; PTC, adrenal cortisol.

### Association Between AST/ALT, INS*PA, and Metabolic Factors

**Figure 1:**
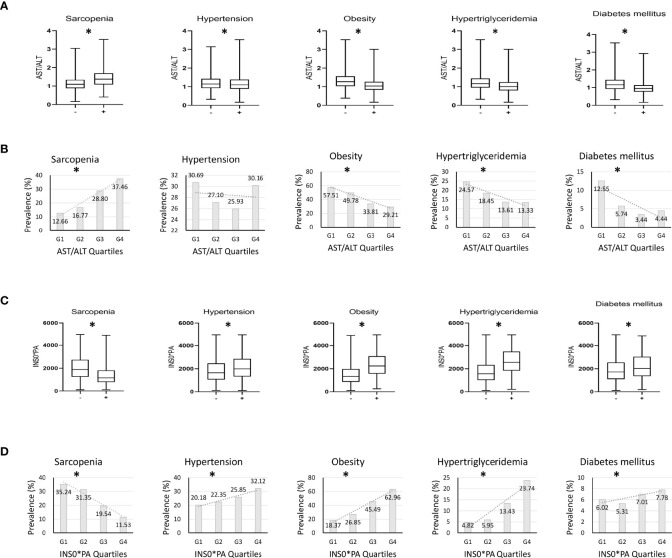
Investigation on whether AST/ALT and INS*PA are affected by metabolic factors. **(A)** Differences in serum aspartate aminotransferase/alanine aminotransferase (AST/ALT) according to the presence of accompanying sarcopenia or metabolic disorders. **(B)** The prevalence of sarcopenia and metabolic disorders according to AST/ALT quartiles. **(C)** Differences in serum fasting insulin*prealbumin (INS*PA) according to the presence of accompanying sarcopenia or metabolic disorders. **(D)** The prevalence of sarcopenia and metabolic disorders according to INS*PA quartiles. **(A, C)** *P < 0.05 between groups; **(B, D)** *P for trend < 0.05.

Among the 4,099 participants, the proportions of sarcopenia, hypertension, obesity, hypertriglyceridemia, and diabetes mellitus were 19.0%, 29%, 50.7%, 21.4%, and 8.7%, respectively. Median AST/ALT levels in the group with metabolic disorders were significantly lower than those in the group without metabolic disorders. In contrast, median AST/ALT was significantly higher in the sarcopenia group than in the non-sarcopenia group (**Figure 1A**, *P* < 0.05, all). **Figure 1B** shows the prevalence of sarcopenia and metabolic disorders according to AST/ALT quartiles. The prevalence of obesity, hypertriglyceridemia, and diabetes mellitus tended to decrease with increasing AST/ALT quartiles, whereas the prevalence of sarcopenia tended to increase with increasing AST/ALT quartiles (*P* < 0.05, all). The prevalence of hypertension was not associated with AST/ALT quartiles. Median INS*PA levels in the group with metabolic disorders were significantly higher than those in the group without metabolic disorders. In contrast, median INS*PA was significantly lower in the sarcopenia group than in the non-sarcopenia group (**Figure 1C**, *P* < 0.05, all). **Figure 1D** shows the prevalence of sarcopenia and metabolic disorders according to INS*PA quartiles. The prevalence of metabolic disorders tended to increase with increasing INS*PA quartiles, whereas the prevalence of sarcopenia tended to decrease with increasing INS*PA quartiles (*P* < 0.05, all).

### Risk Factors for Sarcopenia

**Table 3** shows the results of multivariate analysis by binary logistic regression analysis performed to clarify the risk factors for sarcopenia in all participants, male participants, and female participants, respectively. Variable selection was based on the collinearity between variables, and the most significant variable in similar clinical application biomarkers was selected as the explanatory variable. Binary regression analysis showed that serum AST/ALT >1.14 (OR = 2.247), INS*PA <1,833 μU/ml*g/L (OR = 2.970), FT3 <4.48 pmol/L (OR = 1.313), VitD <18.5 ng/ml (in male participants, OR = 1.817), PTC >336 nmol/L (OR = 1.511), and DBP <81 mmHg (in female participants, OR = 1.250) were independent risk factors for sarcopenia (*P* < 0.05).

**Table 3:** Risk factors for sarcopenia were determined by binary logistic regression analysis.

Risk factors	All participants (*n = *4,099)	*P-*value	Male participants (*n* = 1,471)	*P-*value	Female participants (*n* = 2,628)	*P-*value
	OR (95% CI)		OR (95% CI)		OR (95% CI)	
AST/ALT > 1.14^a^	2.247 (1.883, 2.682)	<0.001	1.967 (1.494, 2.590)	<0.001	2.668 (2.098, 3.393)	<0.001
INS*PA < 1,833^a^ (μU/ml*g/L)	2.970 (2.474, 3.566)	<0.001	3.063 (2.253, 4.165)	<0.001	2.619 (2.073, 3.310)	<0.001
FT3 < 4.48^a^ (pmol/L)	1.313 (1.111, 1.551)	0.001	1.650 (1.264, 2.154)	<0.001	1.223 (0.983, 1.521)	0.071
VitD < 18.5^a^ (ng/ml)	1.257 (1.067, 1.481)	0.006	1.817 (1.388, 2.379)	<0.001	1.131 (0.913, 1.401)	0.258
PTC > 336^a^ (nmol/L)	1.511 (1.280, 1.784)	<0.001	1.498 (1.132, 1.982)	0.005	1.346 (1.087, 1.666)	0.006
UA < 318^a^ (μmol/L)	0.974 (0.823, 1.152)	0.756	1.275 (0.953, 1.707)	0.102	1.083 (0.856, 1.371)	0.504
DBP < 81^a^ (mmHg)	1.110 (0.938, 1.312)	0.224	0.997 (0.761, 1.305)	0.981	1.250 (1.003, 1.558)	0.047

The χ^2^ values were 6.773, 2.091, and 5.339, respectively, for the Hosmer–Lemeshow test (P = 0.561, 0.978, and 0.721, respectively).

a The criterion was the median of all participants.

### The Predictive Value of Indicators for Sarcopenia

**Figure 2:**
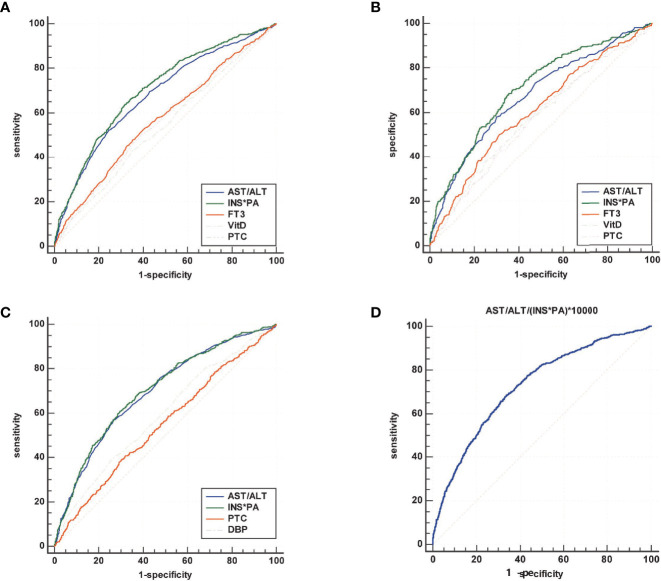
Summary of the results of ROC analysis of various biomarkers for the diagnosis of sarcopenia in all participants **(A)**, male participants **(B)**, and female participants **(C)**, and comprehensive diagnosis in all subjects **(D)**. The area under the ROC curve (AUC) of the INS*PA was the highest (0.705, 0.706, and 0.701, respectively, *P* < 0.05), followed by that of AST/ALT (0.680, 0.675, and 0.695, respectively, *P* < 0.05). In male participants, FT3 and VitD also had certain diagnostic ability, with AUC of 0.606 and 0.583, respectively, but were insignificant in female participants. For PTC, the diagnostic efficacy was limited (0.561, 0.570, and 0.546, respectively, *P* < 0.05). After dividing the two calculated values, the AST/ALT/(INS*PA)*10,000 was obtained, and the AUC of the new calculated value used to diagnose sarcopenia was 0.727.

**Table 4** summarizes the results of ROC analysis of various biomarkers for the diagnosis of sarcopenia in all participants, male participants, and female participants, respectively. The ROC curve is shown in **Figure 2**. Results show that AST/ALT and INS*PA have better diagnostic efficacy for sarcopenia. The AUC of the INS*PA was the highest (0.705, 0.706, and 0.701, respectively, *P* < 0.05), followed by that of AST/ALT (0.680, 0.675, and 0.695, respectively, *P* < 0.05). The cutoff values of INS*PA and AST/ALT in all participants are ≤1,500.16 μU/ml*g/L and >1.35, respectively. In male participants, FT3 and VitD also had certain diagnostic ability, with AUC of 0.606 and 0.583 and with cutoff values of ≤4.43 pmol/L and ≤20.2 ng/ml, respectively, but were insignificant in female participants. For PTC, higher concentration was observed in the sarcopenia group, and the ROC curve showed statistical significance for the diagnosis of sarcopenia, but the diagnostic efficacy was limited (0.561, 0.570, and 0.546, respectively, *P* < 0.05), with a cutoff value of >365.2 nmol/L. After dividing the two calculated values, the AST/ALT/(INS*PA)*10,000 was obtained, and the AUC of the new calculated value used to diagnose sarcopenia was 0.727, with a cutoff value of >7.75, and the sensitivity and specificity were 68.0 and 66.7, respectively (**Figure 2D**).

**Table 4:** The results of ROC analysis of various biomarkers for the diagnosis of sarcopenia.

Biomarkers	All participants (*n = *4,099)	Cutoff values	Sensitivity	Specificity	Male participants (*n* = 1,471)	Cutoff values	Sensitivity	Specificity	Female participants (*n* = 2,628)	Cutoff values	Sensitivity	Specificity
	AUC (95% CI)				AUC (95% CI)				AUC (95% CI)			
AST/ALT	0.680 (0.666, 0.694)	>1.35	52.6	75.4	0.675 (0.651, 0.699)	>1.2	58.2	69.9	0.695 (0.677, 0.712)	>1.35	57.1	73.5
INS*PA (μU/ml*g/L)	0.705 (0.690, 0.718)	≤1,500.16	64.1	68.1	0.706 (0.681, 0.729)	≤1,499.43	68.0	65.1	0.701 (0.683, 0.719)	≤1,478.34	60.7	70.5
FT3 (pmol/L)	0.572 (0.556, 0.587)	≤4.3	48.2	64.6	0.606 (0.581, 0.631)	≤4.43	50.5	68.4	Not applicable		
VitD (ng/ml)	0.539 (0.524, 0.555)	≤16.4	42.9	64.9	0.583 (0.558, 0.609)	≤20.2	57.1	56.3	Not applicable		
PTC (nmol/L)	0.561 (0.546, 0.577)	>365.2	50.9	60.6	0.570 (0.544, 0.595)	>367.4	62.2	51.6	0.546 (0.527, 0.565)	>380.6	39.1	69.8
DBP (mmHg)	Not applicable				Not applicable			0.591 (0.572, 0.610)	≤73	40.4	73.0

AUC, area under the ROC curve.

## Discussion

With the advancement of the global aging process, age-related diseases are becoming a growing concern for the medical community. Generally, this study was conducted to evaluate the relationship between sarcopenia and high serum AST/ALT ratio and low serum INS*PA product levels among elderly Chinese participants from the WCHAT study under the background of China’s aging population. The independent risk factors for sarcopenia were serum AST/ALT >1.14, INS*PA <1,833 μU/ml*g/L, FT3 <4.48 pmol/L, VitD <18.5 ng/ml (in male participants), PTC >336 nmol/L, and DBP <81 mmHg (in female participants). INS*PA and AST/ALT have better diagnostic efficacy for sarcopenia, with AUC of 0.705 and 0.680 and with cutoff values of ≤1,500.16 μU/ml*g/L and >1.35, respectively. After the combination of the two indicators, the AUC for diagnosing sarcopenia could reach 0.727, with a cutoff value of >7.75, and the sensitivity and specificity were 68.0 and 66.7, respectively.

The AWGS modified their diagnostic criteria in 2019 to determine whether subjects have low muscle strength, such as handgrip strength, or low physical performance, using the 6-m walk test. Meanwhile, dual-energy X-ray absorptiometry or bioimpedance was used to assess muscle mass (20). The AWGS diagnostic criteria require hospitals to be equipped with specific instruments that are complex and costly and are not available in all community or primary care hospitals. Therefore, combinations of clinical and serum indicators are needed to predict and assess the risk of sarcopenia, which are measured worldwide as part of regular medical checkups because they can be rapidly, inexpensively, and reproducibly assayed. To our knowledge, this is the first study to predict the risk of sarcopenia using AST/ALT ratio and INS*PA product.

Sarcopenia results in structural or functional defects in skeletal muscles (21). Skeletal muscles are known to contain isozymes of AST, which may be released into the bloodstream following muscle necrosis (22). AST is a clinically accepted biomarker of liver and skeletal muscle injury (23). We found that serum AST activity was higher in patients with sarcopenia, but the difference was not statistically significant compared with those without sarcopenia. Perhaps because AST is widely distributed, the results are influenced by other target organs. Several studies have shown that low serum ALT is associated with lower muscle mass (24) and sarcopenia (25). The results of our research are consistent with the results of other studies. High AST, accompanied by low ALT, predominantly reflects mild-to-moderate skeletal muscle pathology (22, 26), which might imply the presence of incipient sarcopenia. We found a higher AST/ALT in the sarcopenia group, which was a risk factor and a diagnostic marker of sarcopenia. This may explain why an elevated AST/ALT ratio in the sarcopenia group was observed.

Insulin resistance causes loss of muscle tissue, which in turn decreases insulin sensitivity if the glucose supply remains constant and, thus, may lead to a vicious circle and the onset of sarcopenia (27–29). Insulin is involved in the maintenance of muscle mass *via* the p38MAPK and the mTOR/p70S6 kinase pathways, thus suppressing proteolysis (30–32). Insulin deprivation elevates protein breakdown, whereas insulin elevation increases protein synthesis in the setting of amino acid repletion (33). The above may explain why we observed a decrease in median INS levels in the sarcopenia group.

ALB is an indicator of protein metabolic balance in a non-acutely infected individual; however, a half-life of 18 to 21 days does not reflect drastic changes in nutritional status. In a meta-analysis, a significant association was found between low ALB levels and sarcopenia in adults 60 years of age and older (34). PA has a shorter half-life than ALB (16), so its role as a possible biomarker for sarcopenia was evaluated. There are few reports on PA in sarcopenia patients. We found that the sarcopenia group had lower ALB and PA levels. Due to the half-life advantage of PA, we used INS*PA to amplify the difference between the sarcopenia and non-sarcopenia groups and to evaluate its potential as a diagnostic biomarker for sarcopenia. Thus, we observed lower INS*PA levels in the sarcopenia group.

Metabolic syndrome includes hypertension, obesity, hypertriglyceridemia, and diabetes mellitus. Metabolic syndrome causes changes in the concentrations of indicators. To investigate whether AST/ALT and INS*PA are affected by metabolic factors, we obtained from **Figure 1** that they were not and had reversed trends in median levels and prevalence. In addition, there was no statistical significance between AST/ALT and hypertension prevalence; all the others were statistically different. It has been reported that obesity, hypertriglyceridemia, and diabetes mellitus are associated with insulin resistance in adipocytes and hepatocytes, but hypertension is associated with insulin resistance in the vascular endothelium (35), which is why the prevalence of hypertension is not associated with AST/ALT but has to do with INS*PA (9). Lower AST/ALT and higher INS*PA were also able to predict obesity, hypertriglyceridemia, and diabetes mellitus in older subjects.

FT3 stimulates myogenesis and muscle contraction and regulates the metabolism of skeletal muscle cells that decrease with age (36). Mechanistically, VitD directly affected various aspects of muscle function, including myofibrillar protein degradation and impaired calcium transmission in the mitochondria (37). It has been reported that VitD is associated with sarcopenia (38). Nutritional supplementation with VitD attenuates the progression of chronic low-grade inflammatory profile in older sarcopenic persons with mobility limitations (39).

Exposure to excess cortisol plays a negative effect on muscle performance, strength, and muscle quality, which persists even many years after its resolution (40, 41). Glucocorticoids induce atrogin-1 and MuRF-1 in the ubiquitin–proteasome system, thus causing protein degradation of skeletal muscle (42, 43). In our study, higher PTC concentration was observed in the sarcopenia group, but the diagnostic efficacy was limited. The relationship between PTC and sarcopenia needs further study.

We found significant reductions in UA levels in patients with sarcopenia. UA was positively correlated with grip strength and nutritional score (44). In the sarcopenic group, HDL levels increased, while TG and VLDL levels decreased. An inverse relationship was observed between TG and mortality (45). Lower TG levels may be a consequence of poor health status associated with sarcopenia (46). HDL has multiple cardiovascular protective properties, such as anti-inflammatory effects (47). Sarcopenia involves chronic low-grade inflammation, and future research is necessary to validate the relationship between HDL and sarcopenia *via* a cohort study approach. One study in older women reported that appendicular fat-free mass was positively correlated with DBP, which is consistent with our findings (48). We found a higher NPR and a lower LPR in the sarcopenic group than in the non-sarcopenic group.

In conclusion, the current study shows that the independent risk factors for sarcopenia in middle-aged and older patients are serum AST/ALT >1.14, INS*PA <1,833 μU/ml*g/L, FT3 <4.48 pmol/L, VitD <18.5 ng/ml (in male participants), PTC >336 nmol/L, and DBP <81 mmHg (in female participants). Among these risk factors, serum AST/ALT ratio and INS*PA product have stronger effects on sarcopenia. High AST/ALT and low INS*PA are simple biomarkers that can diagnose sarcopenia in older patients, independent of the accompanying metabolic disorders.

## Strengths and Limitations

The present study had several limitations. For one, our measures of smoking/drinking history, activity of daily living, and chronic diseases were based on questionnaires and should thus be interpreted with caution. Second, we used a single measurement of serum. Larger prospective studies are needed to confirm our findings. However, this is the first large-scale study based on a large sample population-based study in West China with complete information on sociodemographics, health conditions, physical examination, anthropometric measurement, and blood sample test, focusing on the general population. We provided potential calculated markers that were cheap and easily accessed for sarcopenia in the clinical setting. More attention should be paid to the levels of AST/ALT and INS*PA in the geriatric population with sarcopenia.

## Data Availability Statement

The original contributions presented in the study are included in the article/supplementary material. Further inquiries can be directed to the corresponding authors.

## Ethics Statement

The studies involving human participants were reviewed and approved by the Ethics Committee of the West China Hospital of Sichuan University (reference: 2017-445). The patients/participants provided their written informed consent to participate in this study.

## Author Contributions

All authors listed have made a substantial, direct, and intellectual contribution to the work and approved it for publication.

## Funding

This study was supported by the National Key R&D Program of China (2020YFC2005600 and 2020YFC2005603) and Sichuan Science and Technology Agency of Sichuan Province (2021YFS0148, 2020YFS0185, and 2019YFS0277).

## Conflict of Interest

The authors declare that the research was conducted in the absence of any commercial or financial relationships that could be construed as a potential conflict of interest.

## Publisher’s Note

All claims expressed in this article are solely those of the authors and do not necessarily represent those of their affiliated organizations, or those of the publisher, the editors and the reviewers. Any product that may be evaluated in this article, or claim that may be made by its manufacturer, is not guaranteed or endorsed by the publisher.
